# Local Anesthetic Infiltration, Awake Veno-Venous Extracorporeal Membrane Oxygenation, and Airway Management for Resection of a Giant Mediastinal Cyst: A Narrative Review and Case Report

**DOI:** 10.3390/jcm14010165

**Published:** 2024-12-30

**Authors:** Felix Berger, Lennart Peters, Sebastian Reindl, Felix Girrbach, Philipp Simon, Christian Dumps

**Affiliations:** 1 Anesthesiology and Operative Intensive Care, Faculty of Medicine, University of Augsburg, 86156 Augsburg, Germany; lennart.peters@uk-augsburg.de (L.P.); felix.girrbach@uk-augsburg.de (F.G.); philipp.simon3@uk-augsburg.de (P.S.); christian.dumps@uk-augsburg.de (C.D.); 2 Department of Thoracic Surgery, Faculty of Medicine, University of Augsburg, 86156 Augsburg, Germany; sebastian.reindl@uk-augsburg.de

**Keywords:** veno-venous extracorporeal membrane oxygenation, mediastinal mass, regional anesthesia, protected mediastinal mass surgery, airway collapse

## Abstract

**Background:** Mediastinal mass syndrome represents a major threat to respiratory and cardiovascular integrity, with difficult evidence-based risk stratification for interdisciplinary management. **Methods:** We conducted a narrative review concerning risk stratification and difficult airway management of patients presenting with a large mediastinal mass. This is supplemented by a case report illustrating our individual approach for a patient presenting with a subtotal tracheal stenosis due to a large cyst of the thyroid gland. **Results:** We identified numerous risk stratification grading systems and only a few case reports of regional anesthesia techniques for extracorporeal membrane oxygenation patients. **Clinical Case:** After consultation with his general physician because of exertional dyspnea and stridor, a 78-year-old patient with no history of heart failure was advised to present to a cardiology department under the suspicion of decompensated heart failure. Computed tomography imaging showed a large mediastinal mass that most likely originated from the left thyroid lobe, with subtotal obstruction of the trachea. Prior medical history included the implantation of a dual-chamber pacemaker because of a complete heart block in 2022, non-insulin-dependent diabetes mellitus type II, preterminal chronic renal failure with normal diuresis, arterial hypertension, and low-grade aortic insufficiency. After referral to our hospital, an interdisciplinary consultation including experienced cardiac anesthesiologists, thoracic surgeons, general surgeons, and cardiac surgeons decided on completing the resection via median sternotomy after awake cannulation for veno-venous extracorporeal membrane oxygenation via the right internal jugular and the femoral vein under regional anesthesia. An intermediate cervical plexus block and a suprainguinal fascia iliaca compartment block were performed, followed by anesthesia induction with bronchoscopy-guided placement of the endotracheal tube over the stenosed part of the trachea. The resection was performed with minimal blood loss. After the resection, an exit blockade of the dual chamber pacemaker prompted emergency surgical revision. The veno-venous extracorporeal membrane oxygenation was explanted after the operation in the operating room. The postoperative course was uneventful, and the patient was released home in stable condition. **Conclusions:** Awake veno-venous extracorporeal membrane oxygenation placed under local anesthetic infiltration with regional anesthesia techniques is a feasible individualized approach for patients with high risk of airway collapse, especially if the mediastinal mass critically alters tracheal anatomy. Compressible cysts may represent a subgroup with easy passage of an endotracheal tube. Interdisciplinary collaboration during the planning stage is essential for maximum patient safety. Prospective data regarding risk stratification for veno-venous extracorporeal membrane oxygenation cannulation and effectiveness of regional anesthesia is needed.

## 1. Introduction

Timely surgical management of symptomatic mediastinal masses is critical, since airway collapse is a potentially fatal complication. Giant mediastinal tumor patients have high rates of perioperative cardiorespiratory complications [1,2].

Central airway occlusion in patients with mediastinal-mass syndrome is a feared complication after anesthesia induction, which therefore warrants the maintenance of spontaneous ventilation, the avoidance of neuromuscular blockade, and airway instrumentation via rigid bronchoscopy [3], especially in pediatric patients [4]. A single-center observational study examining airway patency in 17 adult patients suggested that this is more belief than actual clinical reality [5], but patients were excluded if the patient, the surgeon, or the assigned anesthesiologist refused to participate, creating possible selection bias. This contradiction brings about a dilemma for surgery requiring general anesthesia for median sternotomy in patients with severe compromise of tracheal integrity. Second, severe compression of the trachea due to an outside structure of unknown origin and firmness may render passage with an endotracheal tube impossible. Planning for albeit challenging mechanical oxygenation and ventilation is therefore essential to avoid severe complications. Hemodynamic compromise with right ventricular failure, superior vena cava syndrome (SVCS), cerebral edema due to venous congestion, and infiltration of supra-aortic blood vessels represent the second group of possible life-threatening complications. Although dependent on the specific anatomical characteristics of masses, anesthesia induction is a critical time point for possible hemodynamic decompensation. It is therefore essential to stratify patients with a high degree of safety to find optimal anesthesiologic procedures that provide both maximum patient safety and optimal surgical conditions for resection [6]. We present an individualized approach incorporating the evidence available, including veno-venous extracorporeal membrane oxygenation (V-V-ECMO), with cannulation under regional anesthesia via intermediate cervical plexus blockade (CPB) and suprainguinal fascia iliaca compartment block (FICB) and airway management with videolaryngoscopy and bronchoscopy-guided passage of the endotracheal tube past the stenosis with a 7.0 mm internal diameter (ID) woodbridge tube. To our knowledge, this is the first case report that describes the use of the above-mentioned regional anesthesia procedures and cannulation for V-V-ECMO for elective surgery of a mediastinal tumor, incorporating known risk classifications for an optimal anesthesiologic procedure.

## 2. Narrative Review

In this narrative review, we discuss studies, reviews, and case reports regarding risk stratification of patients with mediastinal tumors and the utilization of regional anesthesia for extracorporeal membrane oxygenation (ECMO) cannulation.

### 2.1. Risk Stratification

Recommended diagnostic studies [7] include computed tomography (CT), echocardiography (especially if symptoms of SVCS are present), lab work according to patient comorbidities, and ultrasound evaluation of blood vessels for cannulation access. Special attention should be paid to patient positioning because patients with SVCS may be unable to lay in a supine position without risking life-threatening hemodynamic compromise. Different scoring systems and clinical features have been proposed to identify patients at high risk of airway collapse [6].

Erdos and Tzanova [7] suggested a classification for possible anesthetic mediastinal mass syndrome, creating categories of safe, unsafe, and uncertain. Clinical warning signs included dyspnea, cough, hoarseness, or syncope; edema in the upper body, especially the face, neck, lids, or larynx; and dilatation of the jugular, arm, and thoracic veins are described as pointers to SVCS. Diagnostics suggested included a CT and dynamic testing, either preoperative awake fiberoptic bronchoscopy or lung function tests with a pneumotachograph [2,8]. In CT, a cross-sectional tracheal area of less than 50% compared to a normal diameter was found to predict a high risk of respiratory complications under general anesthesia. Regarding dynamic testing, a quotient of maximum expiratory flow (MEF) and maximum inspiratory flow (MIF) of below 1 at a vital capacity of 50% (MEF_50_/MIF_50_) was described as highly suggestive of obstruction related to a mediastinal tumor. There was no recommendation for routine bronchoscopy, since its value is highly dependent on the experience of the examining physician and the bronchoscopy itself might induce airway-related complications, such as bleeding or mucosal edema with consecutive complete airway obstruction.

Asymptomatic patients with a cross-sectional tracheal area of more than 50% compared to normal values were classified as safe, while patients with both positive CT and clinical signs of mediastinal mass syndrome were considered unsafe. Patients were assigned to the uncertain category if they had moderate clinical symptoms (with no definition of moderate provided), asymptomatic patients with a tracheal diameter of less than 50% or abnormal dynamic testing, and patients where none of the required diagnostics could be obtained. A clinical grading scale according to tolerance of supine positioning was also proposed to be used, with asymptomatic patients being able to lie supine without any symptoms, patients with mild symptoms experiencing some cough and/or pressure sensations, patients with moderate symptoms tolerating the supine position for short periods (with no cut-off value identified), and patients with severe symptoms not tolerating the supine position for any amount of time.

Regarding SVCS, Qanadly et al. [9] described a radiological classification, based on 16 patients with a helical CT-phlebography, and proposed grading patients into four types described by Stanford and colleagues [10]: type I, with a stenosis of the superior vena cava (SVC) of up to 90%; type II, with a stenosis of 90–99%; type III, with a complete obstruction of the SVC; and type IV, with complete obstruction of both the SVC and one or more major tributaries of the SVC.

Yu et al. [11] proposed a classification system that includes both radiographic and clinical signs of SVCS. All clinical symptoms were most likely to be caused by SVCS and not due to other pathologies such as vocal cord paralysis or compromise of the tracheobronchial tree or heart. Patients are classified as grade 0–5 (Table 1). Incidence estimates and symptom burden are drawn from six studies: Armstrong et al. [12] reviewed medical records of 125 patients with superior vena cava syndrome due to bronchogenic carcinoma, malignant lymphoma, or other tumors compressing the SVC. Yellin et al. [13] reviewed 63 adult and pediatric patients over a 16-year period with bronchogenic carcinoma or lymphoma, while Schraufnagel et al. [14] concluded after retrospective review of 106 adult patients that SVCS rarely constitutes a radiographic emergency. Chen and colleagues [15] retrospectively assessed 45 cases over a twelve-year period, while Rice et al. [16] found that more than half of their 78 included patients with SVCS had pleural effusions. Urruticoechea et al. [17] performed stenting for SVCS in 52 patients with non-small cell lung carcinoma (NSCLC) and described clinical improvement of symptoms.

Potere and colleagues [18] presented two cases of patients with SVCS, where V-V-ECMO was peripherally cannulated in the case of a 40-year-old woman with a 10.7 × 5.9 cm (cm) anterior mediastinal mass and a second 8.1 × 5.6 cm tumor wrapped around the right atrium, and another case of a 28-year-old woman with a mediastinal mass of 8.4 × 12.6 cm that almost completely occluded the SVC, where V-V-ECMO was on standby, with bilateral femoral cannulation completed. The authors recommended using a combination of the grading scales by Qanadly et al. [9] and Yu et al. [11], and additionally a clinical symptom score of SVCS developed by Kishi et al. [19] to support the decision for V-V-ECMO support. This score was developed to risk-stratify eleven patients with SVCS, of which six underwent stent therapy, and uses a combination of neurologic symptoms, laryngopharyngeal or thoracic symptoms, nasal and facial signs or symptoms, and venous dilatation to combine for a maximum score of 10.

Leow et al. [20] and Ramanathan et al. [21] considered patients to be at high risk of mediastinal mass syndrome if they show (I) SVCS, (II) pulmonary artery or right ventricular outflow tract obstruction, (III) airway compression greater than 50% or (IV) if cardiac or great vessels are involved or invaded with a surgical excision and/or reconstruction is deemed likely. Bertini and Marabotti [6] advocated to consider placement of venous and arterial sheaths if one mentioned risk factor from both studies is met and to complete cannulation if more than one risk factor is present. Since achievement of adequate case numbers is difficult, no validated data regarding reliable risk stratification to support decision for completed V-V-ECMO, cannulation (V-V-ECMO standby), or prospective evaluation of proposed risk factors are currently available, regardless of the presence of SVCS. Besides mentioned considerations for optimal anesthesiologic management, V-V-ECMO may yield surgical advantages for optimal tumor resection. The favor seems to tip towards cardiopulmonary bypass or veno-arterial extracorporeal membrane oxygenation (V-A-ECMO) if other mediastinal structures are invaded [22].

### 2.2. Local Anesthetic Infiltration, Regional Anesthesia Techniques, and ECMO-Cannulation

Case reports regarding elective ECMO usage are sparse, with most case reports highlighting ECMO as a rescue therapy for acute respiratory compromise [20,23] or as a bridge for tumor size reduction with chemotherapy [24,25]. Only few cases of combined local anesthetic infiltration with regional anesthesia techniques with planned protective V-V-ECMO implantations for surgery exist. Gourdin et al. [26] described the combination of superficial cervical plexus block (CPB) and a transversus abdominis plane block for V-V-ECMO cannulation for a high-risk rigid bronchoscopy. A small number of studies have evaluated the effectiveness of cervical plexus blockade on central line placement of central venous catheters. Sevim et al. [27] randomized eighty patients to either a superficial or intermediate CPB, with patients in the intermediate CPB group reporting lower pain scores during needle entry, dilatation, and catherization, with no difference during the rest of the procedure. Sheikh and colleagues [28] reported on performing a superficial CPB on four pregnant patients in a case series, while Ciftci et al. [29] reported good performance of superficial CPB in pediatric patients that required hemodialysis catheter placement via the right internal jugular or right subclavian vein.

Cervical plexus blocks are typically used in carotid surgery, superficial neck surgery, and, more recently, thyroid surgery, leading to anesthesia of the anterolateral neck, the clavicle area, and both ante- and retro-auricular areas [30]. Since clinical experience and available studies do not show consistent superiority for the ultrasound-guided technique [31,32], both landmark and ultrasound-guided blocks are widely used. Various studies have debated the exact nomenclature of the different depths of CPB [33,34,35], with the following categorization emerging [36]: the superficial CPB as a subcutaneous infiltration of the cervical plexus in the mid-area of the posterior border of the sternocleidomastoid muscle, the intermediate block with a target area of the space between the sternocleidomastoid muscle and the prevertebral fascia (which involves spread of the local anesthetic to the carotid artery and jugular vein), and the deep CPB as a deposition of local anesthetic between the sternocleidomastoid muscle and a predefined transverse process, most frequently targeting the C2–C4 spinal nerves. Intermediate blocks carry a low risk of phrenic nerve block and are therefore recommended in patients at high risk of respiratory complications. Numerous local anesthetics are used, with some centers recommending prilocaine because of its short half-life compared to other substances [37].

Mahmood et al. [38] and Kusajima et al. [39] reported combined spinal and epidural anesthesia (CSEA) for cesarean sections in patients with mediastinal or neck masses, which served as analgesia for femoral placement of ECMO cannulas and analgesia for the surgery itself. Two case reports describe ilioinguinal–iliohypogastric nerve blocks, either with a continuous [40] or a single-shot technique [41] for patients with localized pain in the femoral cannulation area. Suprainguinal FICB has not been described for femoral cannulation of ECMO or analgesia for ECMO patients. The rationale behind this blockade is the reliable sensory blockade of several nerves passing through the iliac fossa, especially the femoral, obturator, ilioinguinal, and lateral femoral cutaneous nerve [42]. Studies show an increased frequency of sensory loss in the medial thigh and femoral and obturator motor block when ultrasound is used in fascia iliaca blockade [43], especially when a suprainguinal technique is performed [44]. While high volumes of approximately 40 mL of a long-acting local anesthetic such as 0.5% ropivacaine is recommended for total hip arthroplasty, there is a high chance of motor block of both the femoral and obturator nerve [45].

To our knowledge, there are no studies evaluating intermediate CPB or suprainguinal FICB for jugular or femoral ECMO cannulation.

## 3. Clinical Case

**Figure 1:**
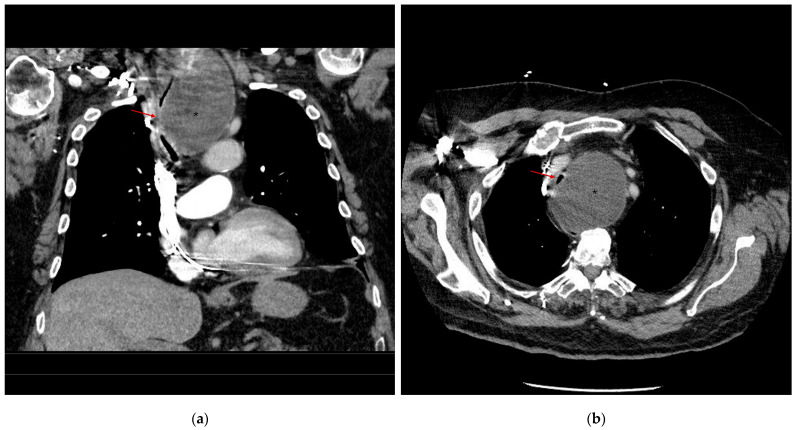
Initial CT imaging of the (**a**) coronal plane and (**b**) axial plane: compression of the trachea (arrow: minimum diameter of 3 mm/subtotal collapse) due to the cyst (*), and a shift in anterior mediastinal structures to the right side of the patient.

A 78-year-old male obese patient with a body mass index (BMI) of 38.3 kg/square meters (kg/m^2^) presented to a community hospital with progressive dyspnea, cough, and stridor that had developed over the course of five weeks. Other comorbidities included non-insulin-dependent diabetes mellitus type II, preterminal chronic renal failure with normal diuresis, grade I arterial hypertension, and low-grade aortic insufficiency. A dual chamber pacemaker was implanted two years prior in a community hospital after a third-grade atrioventricular blockade (complete heart block) was diagnosed. His general physician suspected decompensated heart failure and advised him to present to a community hospital. A CT of the thorax was performed to rule out a pulmonary embolism. It showed a tumor measuring 7.85 (width) × 7.45 (depth) × 10.9 (height) cm, most likely originating from the left thyroid lobe. Subtotal compression of the trachea up to a minimum diameter of 3 mm, the esophagus, the brachiocephalic vein, partial compression of the supra-aortic vessels with subsequent mediastinal shift, and an aneurysm of the ascending thoracic aorta of 43 mm was diagnosed (Figure 1). There was no clinical evidence of SVCS, and the patient was able to rest semi-recumbent at an approximately 30-degree angle.

After imaging, the patient was referred to our university hospital for surgical resection. After interdisciplinary consultation, experienced cardiac anesthesiologists, thoracic surgeons, general surgeons, and cardiac surgeons were assembled to discuss optimal proceedings.

### 3.1. Risk Classification

Due to the subtotal compression of the trachea, partial compression of the supra-aortic vessels, and the mediastinal shift, the patient’s airway was evaluated as high risk of collapse, with uncertain possibility to pass the tracheal stenosis with an endotracheal tube. There were no clinical or radiographic signs of SVCS syndrome, so none of the criteria of the risk classification of Qanadly et al. [9] or Yu et al. [11] were met. In the proposed risk classification by Erdos et al. [7], our patient was classified as unsafe since the prominent stridor, dyspnea, cough, and hoarseness were evaluated as signs of mediastinal mass syndrome and the CT showed a subtotal collapse of the trachea, therefore meeting the criteria of a cross-sectional area of the trachea below 50% compared to normal. Guided by the risk classification of Bertini and Marabotti [6], Leow et al. [20], and Ramathan et al. [21], more than two risk factors were present, with airway compression greater than 50% and involvement of the great vessels present, as they were compressed by the mediastinal tumor. It was decided to opt for protective implantation of a V-V-ECMO under regional anesthesia before anesthesia induction and protected airway management. V-A-ECMO and anesthesia without mechanical ventilation were to be used if airway management failed.

**Figure 2:**
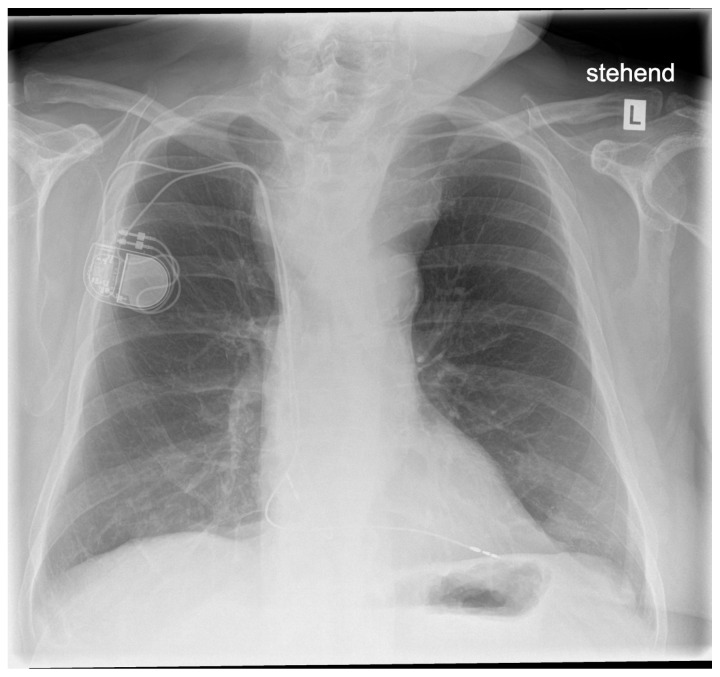
Preoperative chest X-ray with prominent tracheal deviation.

Preoperative evaluation included interdisciplinary clinical evaluation, chest x-ray (Figure 2), lab work, and ultrasound evaluation of blood vessels for ECMO cannulation.

Preoperative lab work showed pathological values for creatinine of 3.61 milligrams/deciliter (mg/dL), an estimated glomerular filtration rate (GFR) according to the Chronic Kidney Disease Epidemiology Collaboration (CKD-EPI) of 15.2 mL/min/1.73 m^2^ and a urea level of 111 mg/dL with normal diuresis, C-reactive protein (CRP) of 1.22 mg/dL, a thyroid-stimulating hormone (TSH) level of 5.66 milliunits/liter (mU/L), and a potassium level of 3.47 millimole/L (mmol/L). Normal lab values included an N-terminal pro-B-type natriuretic peptide (NT-ProBNP) level of 257 picograms/milliliter (pg/mL), and unremarkable complete blood count (CBC) and coagulation parameters. The ultrasound showed small internal jugular veins on both sides (diameter of 0.8 cm on the right side, approximately 0.7 cm on the left side) with no thrombus or other anatomical variation and unremarkable femoral blood vessels, deemed fit for cannulation. No adequate ultrasound window could be obtained for transthoracic echocardiogram (TTE), and after interdisciplinary consultation the decision was made to forego preoperative transesophageal echocardiography (TEE) because of the periprocedural risk of airway collapse. No preoperative dynamic testing of lung function was obtained, since interdisciplinary consultations decided that no matter the result there would be no change in perioperative procedure. Written consent for anesthesia, surgery, and publication of this case report was provided by the patient. He was classified as grade V based on the American Society of Anesthesiologists Physical Status Classification System [46] and grade IV based on the New York Heart Association (NYHA) Functional Classification Scale.

### 3.2. Perioperative Course

After gaining intravenous access, placement of an arterial line, and perioperative antibiotic prophylaxis with two grams of cefazolin, regional anesthesia was performed for optimal patient tolerance of the V-V-ECMO cannulation. Carefully titrated continuous remifentanil (maximum 0.1 microgram/kilogram/minute (µg/kg/min), ideal body weight of 64 kg [47]) was given for supplementary analgesia under supportive oxygen administration of 7 L/minute via an oxygen mask.

A landmark guided right-sided intermediate CPB with 20 milliliters (mL) of 1% prilocaine through a 5 cm 25-gauge needle for optimal tolerance of jugular V-V-ECMO cannulation was applied first. The midpoint of the midline between the transverse process of C6 and the mastoid process was identified and confirmed by two attending anesthesiologists. After penetration of the skin, the needle was advanced perpendicular to the neck, until a loss of resistance and therefore the penetration of the superficial layer of the deep cervical fascia was felt. After a negative aspiration probe, the local anesthetic was injected.

Although the risk of methemoglobinemia was deemed very low with our applied dose of 200 mg (maximum dose for ideal body weight of our patient was 384 mg, with an ideal body weight of 64 kg and a maximum dose of 6 mg/kg bodyweight), methemoglobin levels were monitored through the operative course (Table 2).

For femoral cannulation, using a 5 cm 25-gauge needle, a right-sided ultrasound-guided suprainguinal FICB was performed in a sterile manner. The anterior superior iliac spine was palpated, and a curvilinear ultrasound probe was placed medially in parasagittal orientation and moved caudad until the pelvic brim, the fascia lata, and the deep circumflex iliac artery was located. The needle was advanced from the caudad end of the ultrasound probe with an in-plane technique until a loss of resistance through the fascia iliaca was felt. After negative aspiration and since typical separation or “unzippering” of the fascia iliaca from the iliopsoas muscle was detected and confirmed by two anesthesiologists on ultrasound, we decided to only inject 20 mL of 0.2% ropivacaine to target mostly sensory loss. Following ultrasound evaluation and unsuccessful identification of the fascia lata on the left side, regional infiltration over the left-sided femoral blood vessels with 20 mL of 0.2% ropivacaine was performed for femoral cannulation in case of escalation to V-A-ECMO. The ultrasound guided placement of the venous cannula in the right jugular vein was performed at the same time as the femoral venous cannulation on the right side. A bolus of only 5000 international units (IU) of unfractionated heparin was given to prevent clotting during the cannulation phase with low flow rates. In theory, the coating of the ECMO system allows for circulation for 48 h without any systemic anticoagulation. A right-sided femoral arterial sheath was also placed for rapid escalation to VA-ECMO when needed. Fluoroscopy was used to confirm correct placement of the cannulas. After achieving a flow of two liters per minute, the patient was sufficiently oxygenated and decarboxylated with arterial blood gas analysis showing a partial pressure of arterial oxygen (PaO_2_) of 259 mm of mercury (mmHg) and normocapnia with a partial pressure of arterial CO2 (PaCO_2_) of 35.3 mmHg (for additional values of blood gas analysis, see Table 2), felt well, and was almost apneic, with the stridor completely abated due to the low flow and reduced respiratory rate. Induction of anesthesia was performed with a bolus of 10 µg sufentanil, 100 mg propofol, and 100 mg of rocuronium, with additional preoxygenation via face mask. Sufficient sugammadex doses were readily available for emergency reversal of neuromuscular blockade. A consecutive video-laryngoscopy with a Macintosh blade size 4 showed a Comack–Lehane Grade 1, and a 7.0 mm ID woodbridge tube was placed past the tracheal stenosis with bronchoscopic guidance (Supplementary Video S1: Intubation). A 4.4 mm outer-diameter TRITUBE^®^ together with a Ventrain^®^ (both Ventinova, Eindhoven, the Netherlands) mechanical ventilation system was prepared for usage if the stenosis could not be passed with the woodbridge tube. Additional blood gas analyses showed sufficient oxygenation and decarboxylation, a slightly low potassium level, and stable hemoglobin levels during the operative course (Table 2).

We performed TEE to further confirm correct placement of both V-V-ECMO cannulas with adequate distance between both cannula tips. Recirculation was excluded by post-filter blood gas analysis. Other TEE findings showed the known aneurysm of the ascending aorta of 4.3 cm, grade I aortic insufficiency, a normal left ventricular ejection fraction (LVEF), and no regional wall movement abnormalities. Both V-V-ECMO and mechanical ventilation were continued under intermittent blood gas analysis for the course of the surgery. Near-infrared spectroscopy (NIRS) showed persistent physiological cerebral oxygenation. A continuous norepinephrine–tartrate infusion (initially 0.05 µg/kg/min^−1^, maximum of 0.7 µg/kg/min^−1^, dosage according to the patients estimated ideal body weight of 64 kg, equivalent to 0.0265 µg/kg/min^−1^ and 0.371 µg/kg/min^−1^ norepinephrine base [48,49]) achieved mean arterial pressure (MAP) values over 70 mmHg, deemed adequate because of the patient’s history of grade I arterial hypertension.

A large cyst with fluid content originating from the left thyroid lobe was subsequently resected via a median sternotomy approach with collaboration of thoracic and general surgeons. The capsule was sent for pathological evaluation. After the successful resection, an exit blockade of the ventricular probe due to iatrogenic manipulation of the dual chamber pacemaker prompted surgical revision by cardiac surgery, with the functionless ventricular probe left in place.

Postoperatively, restored normal tracheal anatomy was confirmed via bronchoscopy (Supplementary Video S2: Bronchoscopy: tracheal anatomy after resection), and it was therefore decided to explant the V-V-ECMO in the operating theatre. The patient was then transferred to the intensive care unit and successfully extubated on postoperative day one. Clinical examination showed near normal respiratory function and physical resilience without stridor. Postoperative X-ray imaging showed a regular postoperative status. The new pacemaker aggregate showed normal function at cardiology follow-up on postoperative day one. The ketonemia (maximum ketone level of 3.6 mmol/L), most likely due to sodium glucose-linked transporter 2 (SGLT-2) inhibitor medication, diabetes mellitus type II, and renal insufficiency, was treated with a continuous infusion of insulin and glucose, which led to a normalization of ketone levels (0.2 mmol/L) on postoperative day three. The patient had an otherwise uneventful postoperative course and was transferred to the thoracic surgery ward on day five. The final pathology report showed mixed-follicular thyroid tissue and squamous cell tissue of the capsule with no malignancy. The creatinine level fell back to preoperative values and oral calcium and iodide substitution was started. The patient was released home in stable condition on postoperative day eleven.

## 4. Discussion

The complex interaction between timely resection and maximum patient safety represents a challenge for interdisciplinary teams treating patients with the severe conditions of mediastinal masses. Preoperative selection of patients is challenging, since studies regarding evidence-based risk stratification are still lacking.

As demonstrated by this case report, various elements of anesthesiologic techniques can be applied to guarantee safe proceedings for individual patients suffering from mediastinal masses. Theoretically, our chosen regional anesthesia procedures should cover all relevant anatomic structures, but further cases are needed to confirm this. Our patient described not feeling any pain during cannulation. Single-shot regional anesthesia compared to neuraxial anesthesia like CSEA or epidural anesthesia represent no contraindication for therapeutic anticoagulation and therefore does not interfere with systemic anticoagulation should patients require initiation of cardiopulmonary bypass or need to remain on ECMO for longer than expected. Regional anesthesia might bring additional benefits such as avoiding possible hemodynamic or respiratory decompensation because of sedation and intravenous analgesia, low risk of difficult airway management, or respiratory decompensation during anesthesia induction for intubation and better pain tolerance for awake V-V-ECMO placement in respiratory failure patients in intensive care medicine [50]. Although more evidence is needed, providing local analgesia with local infiltration of the targeted blood vessels would require a multitude of injection sites to cover the area of puncture of the vessels and dilatation with large-bore cannulas. The ultrasound-guided suprainguinal FICB is a standardized technique that requires one injection and in theory allows for sufficient, even spread of the local anesthetic.

Although neuromuscular blockade may not be a prominent threat to airway patency, planning for immediate emergency reversal should be considered. While non-solid tumors like cysts may be easily displaced and passed for with an endotracheal tube, tumors of malignant origin may not, so planning for adequate backup oxygenation is warranted in both cases. Retrospectively, in our specific case, placement of the sheaths for rapid escalation to V-V-ECMO if passage of the stenosed trachea was found to be impossible might have been sufficient.

Utilization of ECMO has spread widely in thoracic surgery, with indications ranging from airway intervention [51] to airway surgery, difficult or impossible single-lung ventilation, mediastinal masses, advanced surgical resections, thoracic emergencies, and lung transplantation [52].

Data regarding outcomes in elective surgery are limited to patients that underwent lung resections for NSCLC [53] with CPB and lung transplantation [54], with trends towards VA-ECMO instead of CPB [55,56]. Prospective studies evaluating outcomes are needed for patients with mediastinal mass syndrome.

## 5. Conclusions and Future Perspectives

Regarding airway collapse, prospective data on the risk of neuromuscular blockade are needed. The authors could not identify any articles regarding risk stratification of solid or, in theory, compressible tumors, so interdisciplinary research including radiographic features could yield a future directive. If positive, radiologic tumor qualities could play a role in risk assessment of patients with mediastinal tumors and risk of mediastinal mass syndrome and could guide clinicians regarding whether cannulation should be completed or whether just the placement of sheaths could be adequate.

Additional prospective data are needed to provide clinicians with validated guidelines for risk stratification of potential airway collapse and mediastinal mass syndrome, since known grading systems are based on expert opinion, small case numbers, or retrospective chart reviews of patient populations such as patients with SVCS. Incidence estimates and symptom burden are drawn from largely retrospective data from heterogenous study populations, so future prospective data should include validating grading systems for symptom relief.

Comparative data of neuraxial blockade versus regional anesthesia for femoral cannulation could identify possible subgroups of patients that benefit from specific procedures. More clinical case reports or series could be a first step in identifying varying success rates of different cervical plexus blocks.

The elective use of protective V-V-ECMO, including local anesthetic infiltration with regional anesthesia techniques in adult high-risk mediastinal mass patients, can be considered a feasible primary approach in centers with a high case load and experienced teams in all participating medical specialties. Preoperative interdisciplinary critical evaluation is essential to optimize the individual approach to patient characteristics and anatomic variations in mediastinal tumors.

## Figures and Tables

**Table 1:** Symptomatic grading system of superior vena cava syndrome by Yu et al. [11].

Grade	Category	Estimated Incidence (%)	Definition
0	Asymptomatic	10	Radiographic superior vena cava obstruction, no symptoms
1	Mild	25	Edema in head or neck (vascular distension), cyanosis, plethora
2	Moderate	50	Edema in head or neck with functional impairment (mild dysphagia, cough, mild or moderate impairment of head, jaw or eyelid movements, visual disturbances caused by ocular edema)
3	Severe	10	Mild or moderate cerebral edema (confusion, obtundation) or mild/moderate laryngeal edema or diminished cardiac reserve (syncope after bending)
4	Life-threatening	5	Significant cerebral edema (confusion, obtundation), significant laryngeal edema (stridor), or significant hemodynamic compromise (syncope without precipitating factors, hypotension, renal insufficiency)
5	Fatal	<1	Death

**Table 2:** Results of blood gas analysis. T0: before V-V-ECMO; T1: after V-V-ECMO cannulation, spontaneous breathing; T2: after anesthesia induction; T3: before V-V-ECMO explantation with minimal flow over the V-V-ECMO system; T4: after V-V-ECMO explantation, directly after transfer to the intensive care unit; T5: three hours after transfer to the intensive care unit.

aBGA Values *	T0	T1	T2	T3	T4	T5
pH	7.41	7.38	7.42	7.35	7.35	7.34
FiO_2_ [%]	21	55	55	40	40	35
PaO_2_ [mmHg]	90	259	238	192	133	106
PaCO_2_ [mmHg]	31	35.8	30.1	37.8	34.1	30.9
SaO_2_ [%]	97	99.9	99.8	99.0	98.4	97.9
HCO_3−_ [mmol/L]	21	21.7	22.1	20.4	18.2	16.0
Base excess [mmol/L]	−4.5	−3.5	−3.5	−4.2	−6.4	−8.7
Glucose [mg/dL]	122	98	108	186	215	189
Ketone level [mmol/L)	-	-	-	-	2.5	3.6
Sodium [mmol/L]	138	139	139	138	139	141
Potassium [mmol/L]	3.1	3.3	3.3	3.5	3.6	4.6
Calcium ionized [mmol/L]	0.98	1.13	1.16	1.15	1.20	1.13
Chloride [mmol/L]	108	108	108	110	110	113
Anion gap [mEq/L]	10	9.3	8.9	7.8	10.9	12.4
Lactate [mmol/L]	2.7	0.9	1.0	1.2	1.6	1.5
Hemoglobin [g/dL]	15.1	12.6	11.7	11.2	12.5	11.3
Hematocrit [%]	-	38.8	36.1	34.4	38.4	34.7
CO Hb [%]	-	1.2	1.3	0.6	0	1.1
Met Hb [%]	-	2.2	1.5	1.2	0.4	0.3

* FiO_2_ = fraction of inspired oxygen, PaO_2_ = partial pressure of arterial oxygen, PaCO_2_ = partial pressure of arterial carbon dioxide, SaO_2_ = arterial oxygen saturation, HCO_3−_ = bicarbonate, anion gap = [sodium] − ([chloride] + [HCO_3−_]), mEq/L = milliequivalents/liter, CO Hb = carboxyhemoglobin, Met Hb = methemoglobin.

## Data Availability

Data are included within the article.

## Supplementary Materials

The following supporting information can be downloaded at: https://www.mdpi.com/article/10.3390/jcm14010165/s1, Video S1: Intubation, Video S2: Bronchoscopy: tracheal anatomy after resection.

## List of Abbreviations

BMI	body mass index
CBC	complete blood count
CPB	cervical plexus block
CKD-EPI	Chronic Kidney Disease Epidemiology Collaboration
cm	centimeter
CRP	C-reactive protein
CT	computed tomography
CSEA	combined spinal and epidural anesthesia
dL	deciliter
ECMO	extracorporeal membrane oxygenation
FICB	fascia iliaca compartment block
GFR	glomerular filtration rate
ID	internal diameter
IU	international units
kg	kilogram
L	liter
LVEF	left ventricular ejection fraction
m^2^	square meter
MAP	mean arterial pressure
MEF	maximum expiratory flow
min	minute
MIF	maximum inspiratory flow
mg	milligram
µg	microgram
mL	milliliter
mm	millimeter
mmHg	millimeters of mercury
mmol	millimole
mU	milliunit
NT-proBNP	N-terminal pro-B-type natriuretic peptide
NYHA	New York Heart Association
NIRS	near-infrared spectroscopy
NSCLC	non-small cell lung cancer
PaO_2_	partial pressure of arterial oxygen
PaCO_2_	partial pressure of arterial carbon dioxide
pg	picogram
SGLT-2	sodium glucose-linked transporter 2
SVC	superior vena cava
SVCS	superior vena cava syndrome
TEE	transesophageal echocardiogram
TSH	thyroid-stimulating hormone
TTE	transthoracic echocardiogram
V-A-ECMO	veno-arterial extracorporeal membrane oxygenation
V-V-ECMO	veno-venous extracorporeal membrane oxygenation
